# Essential tremor with ubiquitinated intranuclear inclusions and cerebellar degeneration 

**DOI:** 10.5414/NP300414

**Published:** 2012-04-18

**Authors:** Elan D. Louis, Pietro Mazzoni, Karen J. Ma, Carol B. Moskowitz, Arlene Lawton, Anthony Garber, Jean-Paul G. Vonsattel

**Affiliations:** 1 GH Sergievsky Center,; 2 Department of Neurology,; 3 Taub Institute for Research on Alzheimer’s Disease and the Aging Brain, College of Physicians and Surgeons,; 4 Department of Epidemiology, Mailman School of Public Health, Columbia University, New York, NY,; 5 Comprehensive Genetics, Molecular Diagnostic Laboratory, Milwaukee, WI,; 6 Department of Pathology and Cell Biology, Columbia University Medical Center and the New York Presbyterian Hospital, New York, NY, USA

**Keywords:** movement disorder, pathology, cerebellum, postmortem, neurodegenerative

## Abstract

Background: Essential tremor (ET), a progressive, age-associated disease, is one of the most common neurological disorders. Yet until recently, there had been few postmortem examinations so that the full range of pathological changes associated with this disease has not been catalogued. Objectives: We report a patient with ET who had a pattern of pathological change which to our knowledge has not previously been reported in ET or another neurological disease. Methods: Clinical-pathological case report. Results: The patient had adult-onset, non-familial, kinetic arm tremor that gradually worsened. Voice and head tremors were also present. The clinical diagnosis was ET. She died at age 102. On postmortem examination, there was severe segmental loss of Purkinje cells, Bergmann gliosis and numerous torpedoes in the cerebellum. The other outstanding change was the presence of neurons in the cerebral cortex and hippocampus that contained an ubiquitinated, nuclear inclusion. These inclusions were not detected in Luxol fast blue/hematoxylin and eosin-stained sections. Conclusions: This ET patient had a pattern of pathological change that has not been reported previously. This case further reinforces the view that ET is likely to be a heterogeneous family of degenerative diseases whose underlying pathological anatomy involves the cerebellum.

## Introduction 

Essential tremor (ET), a progressive, age-associated disease, is one of the most prevalent neurological disorders [1, 2, 3]. Individuals in all age groups, including children [4], may develop ET, although the incidence rises with age and especially with advanced age [5] where the prevalence may exceed 20% [1]. 

The hallmark feature of ET is a kinetic tremor of the arms, yet patients may also exhibit a range of other tremors (e.g., intention, rest), other motor features (e.g., mild gait ataxia), and a range of cognitive deficits including dementia [6]. The tremor itself is often progressive, producing disabilities in basic daily activities such as eating, writing, and body care [7]. First-line medications, of which there are only two, are ineffective in 50% of patients [8], leaving a sizable population of patients untreated and disabled. More recently, physicians have turned to deep-brain stimulation surgery to provide partial relief for severe, pharmacologically-intractable cases [9]. A better understanding of the underlying disease mechanisms is a critical starting point for the development of more effective treatments. 

Although ET shares a number of important clinical features with neurodegenerative diseases [10], until recently, there had been almost no postmortem examinations and the localization of the anatomic pathology was unknown [11]. With intensive efforts to perform detailed postmortems, an understanding of the basic anatomic pathology is just now emerging [11, 12]. In most ET brains studied so far, degenerative changes, including Purkinje cell loss [12], have been observed in the cerebellum; a smaller number of brains have Lewy bodies in the brainstem with relatively normal cerebella [11, 12]. One other case had ubiquitinated intranuclear inclusions in the Purkinje cells [13]. It is becoming apparent that the degenerative changes in ET are heterogeneous, suggesting that ET is not a single disease [10]. The number of published postmortem examinations is still limited (n = 100) and the full spectrum of pathological changes associated with this disease has not been fully catalogued. We report a clinically well-characterized ET patient who had a pattern of pathological change that has not been reported previously in ET or, to our knowledge, in another brain disorder. Thus, the case is instructive, providing potentially useful insights as to the underlying mechanisms of this common neurological disease. 

## Case 

At death, the patient was a 102-year-old, right-handed Caucasian woman with a high school education. Tremor began at age 90 years; she had first noticed it when she used her hands for certain tasks (e.g., pouring water, eating soup with a spoon, putting on make-up). Her tremor worsened over the ensuing 4 years, and at age 94 she was first seen by a neurologist specializing in movement disorders (P.M.) at the Center for Parkinson’s Disease (CPD) and other Movement Disorders at Columbia University Medical Center. She reported that, because of tremor, she was no longer able to eat soup unless she lowered her mouth to the bowl and she could not put on her earrings. Her past medical history was notable for a transient ischemic attack manifested by brief left arm and leg weakness (age 92), atrial fibrillation, hypertension, congestive heart failure, bilateral cataract surgeries, hypothyroidism, arthritis and impaired hearing for which she used a hearing aid. Her thyroid function tests were checked regularly and were normal. There had been no exposure to diphenylhydantoin, cyclosporine or other medications with cerebellar toxicity. She consumed no more than 3 glasses of wine in a typical week. There was no family history of tremor. On examination, there was no postural tremor but kinetic tremor was observed in both arms. The tremor was of moderate amplitude during a variety of tasks (pouring, drinking, drawing spirals) and it resulted in spillage when she used a spoon to drink water. There was no tremor at rest and no features of parkinsonism or dystonia. The remainder of the neurological examination was normal, except for mild difficulty with tandem gait. There was no dysarthria, intention tremor, dysdiadochokinesia, or frank ataxia. A clinical diagnosis of ET was assigned. Low-dose primidone was initiated (25 mg b.i.d.), resulting in excellent tremor control over the ensuing weeks. 

Between the ages of 94 and 100, she was seen 8 times at the CPD by the same neurologist (P.M.). She continued to report that her hand usually trembled when she held a pen to write and that her tremor often embarrassed her. At age 95, she was enrolled in a clinical epidemiological study of ET at Columbia University [14] and as a potential brain donor into the Essential Tremor Centralized Brain Repository [15]. A brief cognitive screen, the Telephone Interview for Cognitive Status [16] was administered; the score, 35 of 41, was above the range considered to be indicative of dementia (< 31). On a Folstein Mini-Mental Status Examination [17], she similarly scored 29 out of 30. A videotaped tremor examination at that time revealed no rest tremor, minimal postural tremor of the left arm only, tremor while pouring (mild-to-moderate on the right and moderate on the left), tremor while using a spoon (moderate bilaterally), tremor while drinking from a cup (moderate on the right and mild on the left), tremor during the finger-nose-finger maneuver (mild-to-moderate on the right and moderate on the left), and tremor while drawing spirals (mild on the right and moderate on the left). There was a mild and intermittent side-side (“no-no”) head tremor but no voice tremor. The finger-nose-finger tremor had a mild intentional component (left greater than right); an intention tremor of the head was also noted when she drank water from a cup. Rapid alternating movements, facial expression and speech were normal and there was no dystonia. Based on these data, a senior movement disorders neurologist (E.D.L.) diagnosed ET using standardized, published clinical research criteria (moderate or greater amplitude kinetic tremor observed during 3 or more activities or a head tremor, in the absence of Parkinson’s disease) [18]. 

At age 98, during her visit to the CPD, she reported an increase in hand tremor such that she could no longer hold a tea cup; primidone was increased to 50 mg b.i.d., with some resultant tremor reduction. At age 100, a voice tremor was first noted on examination; tremor in her hands had also increased and there was now an intentional component, in addition to kinetic tremor, on the finger-nose-finger maneuver. At the time of her final visit (age 100), she used walker due to declining confidence in her gait; her advanced age, bilateral hip replacements, and a possible hair-line fracture adjacent to the hip replacement were the major contributors to her tentative gait. There were no cognitive complaints and she continued to live independently and swam daily for exercise. She died at home at age 102 of cardiopulmonary arrest. 

## Postmortem brain examination 

The brain, which weighed 1,054.3 g, was placed on ice 2.5 h after death. At the New York Brain Bank, Columbia University, external examination (J.P.G.V.) revealed mild (1+) frontal atrophy; in addition, the amygdala was mildly atrophic. Otherwise, the brain was grossly unremarkable. 

As described [12, 19], blocks were taken from standardized brain regions and embedded in paraffin; 7 mm-thick sections were stained with Luxol fast blue counterstained with hematoxylin-eosin (LH&E). Additional sections from selected blocks were stained with modified Bielschowsky silver stain, and others, with antibodies directed against α-synuclein (1 : 40, Leica, Buffalo Grove, IL, USA) (including cerebral cortex, hippocampal formation, globus pallidum, putamen, amygdala, midbrain with substantia nigra, pons with the locus ceruleus, medulla with the dorsal vagal nucleus, and olfactory bulbs), β-amyloid (1 : 400, Biocare Medical, Concord, CA, USA), hyperphosphorylated τ (AT8) (1 : 200, Thermoscientific, Rockford, IL, USA), and glial fibrillary acidic protein (GFAP) (Ventana, Tucson, AZ, USA) proteins. The hippocampal formation and the cingulate gyrus were stained with antibodies directed against Tar-DNA binding protein-43 (TDP-43) (1 : 2,000, Protein Tech Group, Chicago, IL, USA), Fused in Sarcoma (FUS) (1 : 500, Millipore, Billerica, MA, USA) and p62 (1 : 500, Millipore). The following selected blocks were stained with antibodies directed against ubiquitinated proteins (1 : 300, Dako, Carpinteria, CA, USA): superior frontal cortex; posterior frontal cortex; parietal cortex; calcarine cortex; hippocampal formation with lateral geniculate body and tail of caudate nucleus; caudate, putamen, and nucleus accumbens; globus pallidus and putamen with claustrum; cerebellum; subthalamic nucleus with anterior thalamus; anterior hippocampal formation; and pituitary gland. Using ubiquitin-stained sections of several brain regions, we quantified the number of inclusions: in five 630× fields, we counted the number of inclusions per neuron with visible nucleolus. This was expressed as a percentage. A 3 × 20 × 25 mm standard parasagittal cerebellar cortical block was immersion-fixed in 10% buffered formalin. Torpedoes in the entire LH&E section and another entire Bielschowsky-stained section were counted [12]. Purkinje cells in 15 randomly-selected 100× LH&E stained fields of the standard cerebellar section were counted. The average of these 15 counts was reported as the mean number of Purkinje cells per 100× field. Bergmann cells were assessed using a GFAP-immunostained section. A semiquantitative rating of the appearance of the basket cell plexus surrounding Purkinje cell bodies throughout the Bielschowsky preparation was applied using the following scale: 0 (few or no discernible processes); 1 (sparse number of processes); 2 (moderate number of processes); and 3 (dense tangle of processes), as described [20]. 

**Figure 1:**
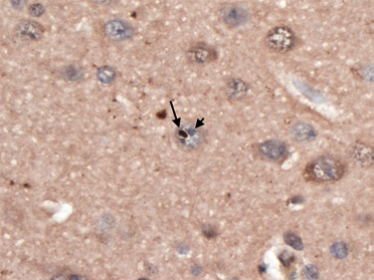
Superior frontal cortical neuron with an ubiquitinated, nuclear inclusion (long arrow) adjacent to the nucleolus (short arrow). Ubiquitin × 630.

**Figure 2:**
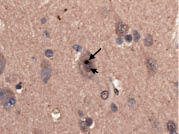
Superior frontal cortical neuron with an ubiquitinated, nuclear inclusion (long arrow) adjacent to the nucleolus (short arrow). Ubiquitin × 630.

**Figure 3:**
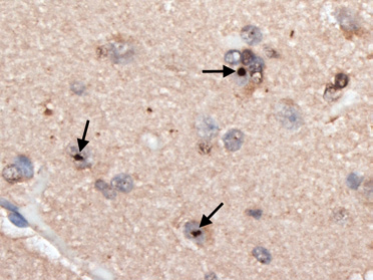
Insular cortex showing several ubiquitinated, nuclear inclusions (arrows). Ubiquitin × 630.

**Figure 4:**
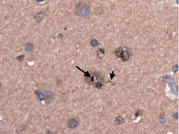
Superior frontal cortical neuron with an ubiquitinated, nuclear inclusion (long arrow). A dystrophic neurite is also seen (short arrow). Ubiquitin × 630.

The neuronal density was normal throughout the cerebral cortex. The outstanding change consisted of the presence of scattered neurons that contained an ubiquitinated, nuclear inclusion (Figure 1, Figure 2). These neurons, which tended to form groups (Figure 3) and predominated within the deep cortical layers, were found throughout the cerebral cortex. In addition, occasional ubiquitinated dystrophic neurites were seen especially in the vicinity of the neurons including nuclear inclusions (Figure 4). These inclusions were not detected in LH&E-stained sections, or in sections subjected to AT8 antibodies directed against phosphorylated t, or to antibodies directed against α-synuclein aggregates, or against amyloid deposits. Although rare, neurons with an ubiquitinated nuclear inclusion were also found within the CA4, CA2-3, and CA1 regions of the hippocampus and parahippocampal gyrus. Neither the neo- nor the paleostriatum showed ubiquitinated neuronal nuclear inclusions. These inclusions were not observed in astrocytes or oligodendrocytes. Hippocampal and cingulate sections, which were subjected to TDP-43 antibodies, did not reveal any nucleo-cytoplasmic translocation of labeling or neuropil skeins; the same sections, subject to FUS did not reveal any significant abnormality. The sections from the same regions subject to p62 antibodies confirmed the presence of ubiquitinated neuronal nuclear inclusions and that of scattered dystrophic neurites. Notably a few ubiquitinated neuronal nuclear inclusions were found within the CA4, CA2-3, CA1 and parahippocampal gyrus, and within the cingulate gyrus. 

Using ubiquitin-stained sections of several brain regions, we quantified the number of inclusions by counting, in five 630× fields, the number of inclusions per neuron with visible nucleolus. This was expressed as a percentage. The results were as follows: superior frontal cortex (24.3%), posterior frontal cortex (11.8%), superior parietal cortex (18.5%), primary visual cortex (3.2%), hippocampus (CA4) (7.4%), hippocampus (CA2-3) (27.5%), hippocampus (CA1) (2.9%), and parahippocampal gyrus (12.5%). By comparison, we used the same method to quantify the number of inclusions in 20 normal control brains (mean age = 85.2 y, range = 74 – 104 y). Inclusions were quantified in the superior frontal cortex, and CA1, 2-3 and 4 regions of the hippocampus; we did not find any (0% in each brain region). 

The frontal, parietal, and occipital neocortex was without argyrophilic neuronal tangles, without AT8-labeled neurons, and without visible amyloid deposits. Rare argyrophilic neuronal tangles involved the upper layers of the entorhinal cortex, parahippocampal, and occipitotemporalis gyri, where they were outnumbered by AT8-labeled neurons that were intermingled with neuropil threads. The temporal pole exhibited scattered AT8-labeled neurons and threads. Rare neuropil threads were seen within the prefrontal or parietal region. The Braak & Braak [21] Alzheimer’s disease (AD) stage was IV. The sections subjected to α-synuclein antibodies did not reveal any abnormal aggregates. 

**Figure 5:**
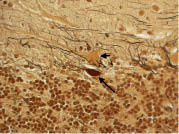
Cerebellar cortex. A torpedo (long arrow) is seen adjacent to a Purkinje cell body (short arrow). Bielschowsky × 200.

**Figure 6:**
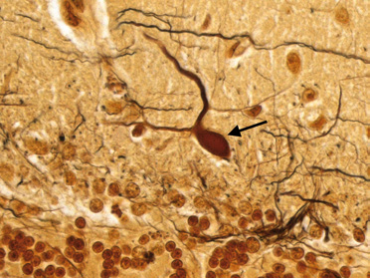
Cerebellar cortex. An abnormal swelling of the Purkinje cell dendritic arbor is evident (arrow). Bielschowsky × 630.

In the cerebellum, there was an excess of torpedoes, noted especially on Bielschowsky stained sections. There were 5 torpedoes in the entire LH&E stained cerebellar cortical section (published value from elderly control brains = 1.7 ± 1.4, and value from ET brains with cerebellar changes = 12.6 ± 7.9) [12] and 17 torpedoes in the entire Bielschowsky stained cerebellar cortical section (Figure 5) (published value from normal elderly brains = 3.3 ± 7.3, and value from ET brains with cerebellar change 19.6 ± 14.5) [12]. Abnormal swellings of the Purkinje cell dendritic arbor were also evident (Figure 6). The segmental loss of Purkinje cells was severe in many regions (Figure 7A, B). The mean number of Purkinje cells per 100× field was 4.3 (respective published value from elderly normal brains = 9.6 ± 3.4, and from ET brains with cerebellar changes = 6.6 ± 2.4) [12]. The Bergmann gliosis was segmental, the severity of which varied from marked to mild with interposed parts that were apparently free of gliosis. The semiquantitative rating of the appearance of the basket cell plexus surrounding Purkinje cells = 2 (published mean value from elderly control brains = 1.39, and mean value from ET brains with cerebellar changes = 1.84) [12], indicating the likely presence of reactive structural reorganization of basket cell processes in response to Purkinje cell loss (Figure 8). The dentate nucleus was normal. Ubiquitinated, nuclear inclusions were not found in the cerebellum. 

Postmortem testing of frozen brain tissue was negative for Fragile X Tremor Ataxia Syndrome (FXTAS) (both 29 repeats; normal < 55 repeats), Spinocerebellar Ataxia (SCA) 1 (both 29 repeats; normal < 45 repeats), SCA 2 (22 and 26 repeats; normal < 32 repeats), SCA 3 (both 23 repeats; normal < 45 repeats), SCA 6 (both 11 repeats; normal < 19 repeats), and SCA 7: (10 and 12 repeats; normal < 36 repeats). 

## Discussion 

The patient we report had a 12-year history of ET and, on postmortem examination of the brain, had ubiquitinated, neuronal nuclear inclusions throughout the cerebral cortex and hippocampus as well as severe segmental loss of Purkinje cells, Bergmann gliosis, and numerous torpedoes in the cerebellum. 

Neuronal intranuclear inclusion disease is a rare neurodegenerative disorder with a heterogeneous clinical picture that can include parkinsonism, cerebellar signs, pyramidal tract signs, and dementia accompanied by the diffuse presence of ubiquitin positive inclusions in the brain [22, 23, 24]. In addition, neuronal loss is often present, though not in the brain regions in which inclusions are present. In marked contrast to published cases, the ubiquitinated inclusions in our case were not detected in LH&E or H&E-stained sections [22, 23, 24]. Hence, this patient had a pattern of pathological change that has apparently not been reported previously in the literature. Furthermore, we are unaware of any reports of patients with diffuse neuronal intranuclear inclusions and a clinical picture of ET. 

The observation of ubiquitinated-protein inclusions is one of the hallmarks of neurodegeneration. Ubiquitinated-intranuclear inclusions are found in a variety of neurodegenerative diseases including FXTAS, SCAs, and Huntington’s disease; however, in these disorders inclusions are accompanied by marked additional changes on postmortem [25]. The mechanism of accumulation of these protein inclusions is not clear but it may be tied with the attempted clearance and detoxification of damaged proteins. 

In published cases of neuronal intranuclear inclusion disease, the inclusions were often observed in morphologically well-preserved neurons, and quantitative studies have demonstrated this inverse correlation; thus, inclusions seem to be numerous in brain regions without obvious neuronal loss and infrequent in brain regions in which neuronal depletion is severe [24]. Similarly, in polyglutamine repeat diseases, this discrepancy between inclusion formation and neuronal death has also been reported, leading some investigators to suggest that the inclusions might be neuroprotective [24, 26, 27, 28]. The same pattern was observed in the present case, with inclusions detected in brain regions with no obvious neuronal depletion (cerebral cortex) and the absence of inclusions in the cerebellum, where Purkinje cell loss was marked. 

The role in this case that the inclusions played in producing the clinical symptoms and signs is not entirely clear, as they involved several cerebral cortical regions as well as the hippocampus. However, they seemed to be more abundant in the primary sensorimotor cortex, which seems to be involved in the propagation of tremor in ET [29], than they were in the primary visual cortex, which plays no role in tremor generation or propagation in ET. However, clinical-pathological correlations should in general be regarded with caution. In FXTAS, a disorder for which ubiquitinated-intranuclear inclusions are a clear pathological hallmark, the regional distribution of inclusions often does not correlate with the presence or progression of clinical features [30]. Similarly, in HD Like-2, another disorder for which ubiquitinated-intranuclear inclusions occur, the regional distribution of inclusions within the brain does not necessarily correlate with the clinical symptomatology [31]. 

Our patient had a clinical diagnosis of ET, independently assigned by two neurologists specializing in movement disorders using both clinical criteria as well as stringent research criteria for ET. Clinical features that were consistent with ET were (1) the presence in both upper limbs of kinetic tremor of moderate amplitude on multiple tasks, in the absence of parkinsonism or dystonia, (2) a mild intentional component to arm tremor, (3) the greater amplitude of kinetic than postural tremor, (4) the gradual yet progressive worsening of limb tremor with time, (5) the later development of tremor in cranial structures (“no-no” head tremor and voice tremor), (6) absence of dysarthria, dysdiadochokinesia, or marked ataxia, and (7) clinical response to primidone. There was no family history and a relatively old age of onset, but both sporadic ET and onset of ET in advanced age are well-known to occur [5]. Hence, there was nothing clinically atypical about this case. 

Our patient did not have FXTAS as the genetic testing was negative. Furthermore, an ET-like phenotype is very rare in that disorder [32]. Against a diagnosis of SCA is the clinical presentation (absence of dysarthria, dysdiadochokinesia, or marked ataxia), negative genetic test results, and postmortem findings (e.g., absence of: pontine atrophy, neuronal loss in inferior olivary nucleus or more complete loss of Purkinje cells). 

The study of the pathological anatomy of ET is in its infancy. Postmortem studies over the past several years have indicated that a variety of different types of degenerative changes occur in the ET brain, indicating that ET is likely to be a family of diseases with an overlapping clinical phenotype rather than a single clinical-pathological entity [11, 12]. In most ET brains, degenerative changes, including Purkinje cell loss [12], have been observed in the cerebellum. A smaller number of brains have Lewy bodies in the brainstem (esp. locus ceruleus) with relatively normal cerebella, although given the direct synaptic connections between neurons of locus ceruleus and Purkinje cells, it has been suggested that cerebellar dysfunction may be occurring in these cases as well [11, 12]. One reported ET case also had ubiquitinated intranuclear inclusions in the Purkinje cells [13]. We now report an ET patient who had both cortical nuclear inclusions as well as degenerative changes in the cerebellum. 

Aside from the unique pathological features, the case is of additional import because it (1) further reinforces the view that ET is likely to be a heterogeneous family of diseases rather than a single clinical-pathological entity, (2) reinforces the notion that ET is disease characterized by neurodegenerative changes (i.e., cell loss, neuronal inclusions), and (3) provides additional evidence that the underlying pathological anatomy in ET involves the cerebellum. 

## Acknowledgments 

R01 NS42859 from the National Institutes of Health (Bethesda, MD); the Parkinson’s Disease Foundation (New York, NY, USA). 

**Figure 7:**
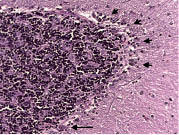
A. Cerebellar cortex. Segmental loss of Purkinje cells with Bergmann gliosis. A single Purkinje cell body is seen (long arrow) but there are otherwise no visible Purkinje cells. Areas of Bergmann glia are marked by short arrows. LH&E × 200.

**Figure 7:**
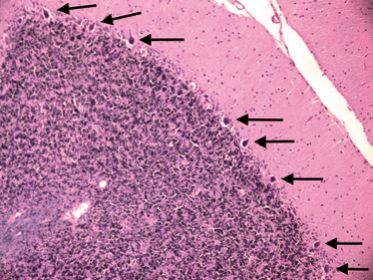
B. Cerebellar cortex. The most preserved region of the Purkinje cell layer in this case. This may be compared with Figure 7A. More Purkinje cells (arrows) are identifiable than in Figure 7A, although there is still some patchy loss of Purkinje cells and Bergmann gliosis. LH&E × 100.

**Figure 8:**
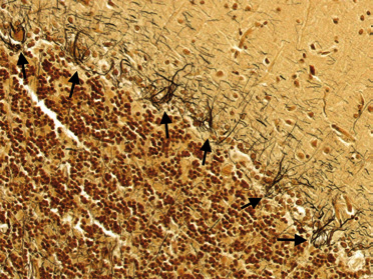
The semiquantitative rating of the appearance of the basket cell plexus surrounding Purkinje cells = 2, indicating the likely presence of reactive structural reorganization of basket cell processes in response to Purkinje cell loss. Several basket cell plexuses are marked by arrows. Bielschowsky × 200.
